# Circulating MAIT cells in multiple sclerosis and amyotrophic lateral sclerosis

**DOI:** 10.3389/fimmu.2024.1436717

**Published:** 2024-07-23

**Authors:** Davide De Federicis, Claudia Bassani, Rosaria Rita Chiarelli, Federico Montini, Antonino Giordano, Federica Esposito, Nilo Riva, Angelo Quattrini, Vittorio Martinelli, Massimo Filippi, Cinthia Farina

**Affiliations:** ^1^ Institute of Experimental Neurology and Division of Neuroscience, San Raffaele Scientific Institute, Milan, Italy; ^2^ Neurology Unit, Division of Neuroscience, Istituto di Ricovero e Cura a Carattere Scientifico (IRCCS) San Raffaele Scientific Institute, Milan, Italy; ^3^ Neurophysiology Service, Istituto di Ricovero e Cura a Carattere Scientifico (IRCCS) San Raffaele Scientific Institute, Milan, Italy; ^4^ Vita-Salute San Raffaele University, Milan, Italy

**Keywords:** amyotrophic lateral sclerosis, blood, CD161, MAIT cells, progressive multiple sclerosis

## Abstract

Neurological disorders, including multiple sclerosis (MS) and amyotrophic lateral sclerosis (ALS), may be associated with alterations in blood cell composition and phenotype. Here, we focused our attention on circulating mucosal-associated invariant T (MAIT) cells, a CD8^+^ T cell memory population expressing the invariant Vα7.2 region in the T cell receptor and high surface levels of the CD161 marker. Transcriptomics data relative to peripheral blood mononuclear cells (PBMC) highlighted downregulation of CD161 and other MAIT-associated markers in progressive MS and not relapsing remitting (RR)-MS when gene expressions relative to each disease course were compared to those from healthy controls. Multiparametric flow cytometry of freshly isolated PBMC samples from untreated RR-MS, primary or secondary progressive MS (PP- or SP-MS), ALS and age- and sex-matched healthy controls revealed specific loss of circulating CD8^+^ MAIT cells in PP-MS and no other MS courses or another neurological disorder such as ALS. Overall, these observations point to the existence of immunological changes in blood specific for the primary progressive course of MS that may support clinical definition of disease.

## Introduction

Multiple sclerosis (MS) is a chronic disorder of the central nervous system presenting with distinct clinical courses (1, 2). Most of MS patients develops the relapsing-remitting (RR) form of disease, where episodic neurological disability due to inflammatory events within the central nervous system is followed by partial or total remission (1). After several years these patients commonly evolve to a progressive phase called secondary progressive (SP)-MS characterized by steady, gradual neurologic deterioration (1). About 15% of MS patients experience a progressive course from onset and are referred to as primary progressive (PP)-MS (1). Despite advances in applications of magnetic resonance imaging (MRI), radiological findings in progressive MS may overlap with RR-MS or other neurological diseases, such as neuromyelitis optica, central nervous system neoplasms or infections (3, 4). Thus, the identification of pathogenic features associated with distinct clinical courses represents an unmet need to better support definition of progression in MS. In this regard, we previously demonstrated that peripheral blood mononuclear cells (PBMC) bear specific transcriptional dysregulations in several genes and pathways in different MS phases and clinical courses (5–8).

Some recent literature suggests the involvement of mucosal-associated invariant T (MAIT) cells in neurological disorders, including MS (9–11) and amyotrophic lateral sclerosis (ALS) (12, 13). This CD8^+^ T cell memory population expresses the invariant Vα7.2 region in the T cell receptor and high surface levels of the CD161 marker, releases inflammatory cytokines such as IL-17 upon recognition of microbial products by means of the non-polymorphic MHC-related protein 1 (14), and is mostly located in tissues, representing only 1-4% of all T cells in peripheral blood (15).

Here, we demonstrate selective reduction of circulating CD8^+^ MAIT cells in PP-MS and no other phenotypes of MS or ALS.

## Materials and methods

### Human subjects

Investigations were conducted according to the principles expressed in the Declaration of Helsinki and after approval of the study by the Ethics Committee of Ospedale San Raffaele. MS subjects were diagnosed according to McDonald criteria (16) and classified according to disease course (1), while ALS diagnosis was based on the revised El Escorial criteria (17). At the time of blood sampling RR-MS patients were in the remission phase, RR-MS and PP-MS subjects were treatment naïve, while SP-MS patients had not received any therapy for the last six months. After signing of the informed consent peripheral blood was drawn from neurological patients and age- and sex-matched healthy controls (**Supplementary Figure 1**).

### Peripheral blood mononuclear cell preparation and flow cytometry

PBMC were isolated by a discontinuous density gradient (Lymphoprep, Nycomed, Oslo, Norway) as already described (7, 8). Viable cells were counted by Trypan Blue (Sigma-Aldrich, Milan, Italy) exclusion. Freshly prepared cells were stained with FITC-labeled anti-human CD45RO (UCHL1, Biolegend), PE-labeled anti-human CD161 (HP-3G10, Biolegend), PerCP-labeled anti-human CD4 (SK3 Biolegend), PeCy7-labeled anti-human CD197 (CCR7, BD), APC-H7-labeled anti-human CD8 (SK1, BD Biosciences), APC-labeled anti-human TCR Vα7.2 (3C10, Biolegend) and Pacific Blue-labeled anti-human CD3 (UCHT1, Biolegend). Samples were acquired at FACSCanto II using FACS DIVA software (all from BD Biosciences). Data were analyzed by FlowJo software (FlowJo LLC). Thresholds were set on FMO and isotype controls, and distinct gating strategies were applied as depicted in **Supplementary Figures 2** and **5**.

### Transcriptomics analysis

The human PBMC transcriptomics dataset analyzed in this study was already published (5–8) and deposited at EBI Array express database (ID: E-MTAB-4890). This dataset was generated by Illumina Human Ref-8 v2 microarrays and included PBMC transcriptomes of 23 PP-MS, 21 SP-MS, 53 RR-MS and 40 healthy controls (HC). Raw data were processed in R using Limma package. Background was subtracted by nec method, data were normalized using cubic spline procedure, and batch effects were corrected by Combat. To reduce variability and normalize the distributions, the dataset was processed using a logarithmic scale base 2.

### Statistical analysis

Normality of data distribution was assessed by D’Agostino and Pearson statistics. Unpaired t-test (in case of normal distribution) or non-parametric Mann–Whitney U test (in case of non-normal distribution) was applied to compare means between independent groups. Welch’s correction was applied to the t-test in case of normality with unequal variances. Sex distribution between groups was evaluated by Fisher’s test. All raw *p*-values were two-sided and subjected to a significance threshold of 0.05. Statistical analyses were performed with GraphPad Prism and R softwares.

### Results

**Figure 1:**
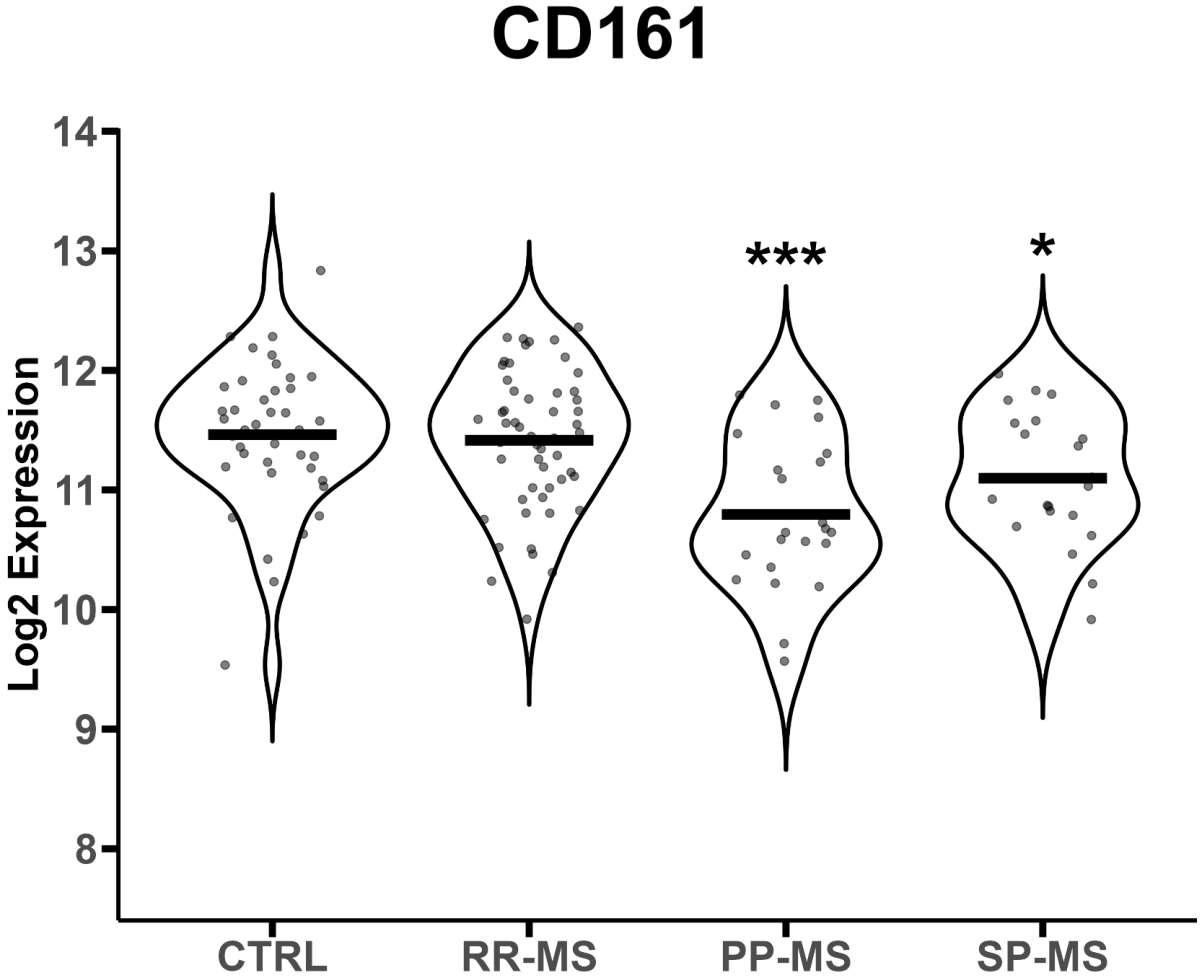
CD161 gene mRNA levels are down-regulated in progressive MS. CD161 gene transcript levels (reported in Log2 expression) were evaluated in PBMCs from 40 healthy controls (HC), 23 PP-MS, 21 SP-MS and 52 RR-MS patients. Group comparisons were evaluated by unpaired t-test with Welch’s correction in case of unequal variances. * *p*<0.05, *** *p*<0.001.

**Figure 2:**
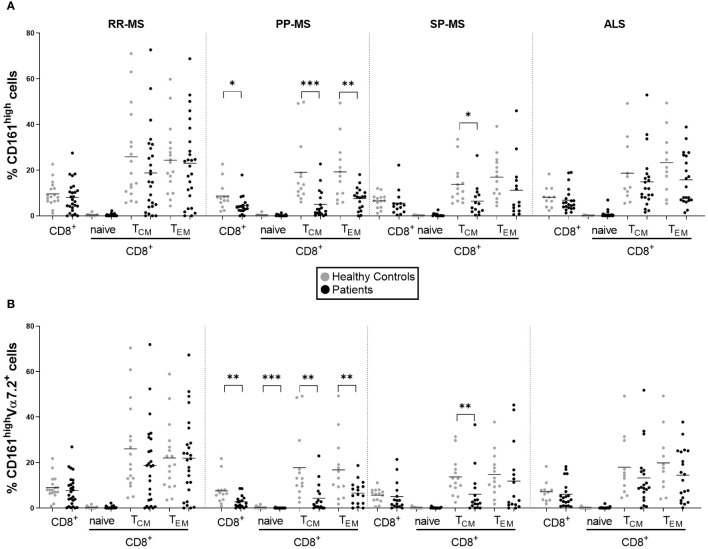
Circulating MAIT cells are reduced in primary progressive MS and no other MS forms or ALS. The frequencies of CD161^high^ and CD161^high^Vα7.2^+^ MAIT cells among total CD8^+^ T cells and the respective central memory (T_CM_, CD45^+^CCR7^+^), effector memory (T_EM_, CD45RO^+^CCR7^-^) and naïve (CD45RO^-^) subsets, were evaluated by multiparametric flow cytometry in 26 RR-MS (mean age 39.81), 19 PP-MS (mean age: 53.61), 14 SP-MS (mean age: 53.93) and 21 ALS patients (mean age: 58.19) (black circles) and compared to age- and sex-matched healthy controls (grey circles) **(A, B)**. Group comparisons were evaluated by unpaired t-test with Welch’s correction in case of unequal variances or Mann-Whitney test in case of non-normal distribution. * *p*<0.05, ** *p*<0.01, *** *p*<0.001.

We analyzed publicly available gene expression data (E-MTAB-4890) relative to PBMC of RR-MS, PP-MS, SP-MS patients and HC, and tested whether MAIT cell markers were differentially expressed in any disease group compared with HC. *CD161* gene transcripts were significantly down-regulated in the PBMC of progressive forms of MS and not RR-MS (**Figure 1**). Furthermore, several other genes associated with MAIT cells, such as RORγt, CCR6, CXCR6 and IL-7 receptor (14), were also specifically less represented in PBMC of progressive MS patients (**Supplementary Figure 3**). We then moved to CD161 protein validation by multiparametric flow cytometry on T cells in order to evaluate whether its transcriptional down-regulation was mirroring changes in circulating CD161^+^ T cell populations in novel cohorts of untreated RR-, PP- and SP-MS patients compared to age- and sex-matched healthy controls (**Supplementary Figure 1**). The investigation was also extended to samples from patients with ALS. Parallel analyses of CD4^+^ CD161^+^, CD8^+^ CD161^int^ and CD8^+^ CD161^high^ T cells restricted CD161 signal loss to the CD8^+^ CD161^high^ subset in PP-MS (**Supplementary Figures 2**, **4**; **Figure 2A**). Notably, ALS patients displayed a significant increase of CD4^+^CD161^+^ and CD8^+^CD161^int^ T cell populations (**Supplementary Figure 4**). Further stratification of the CD8^+^ population into naïve (CD45RO^-^), effector memory (T_EM_, CD45RO^+^CCR7^-^) and central memory (T_CM_, CD45RO^+^CCR7^+^) phenotypes showed a slight rise in CD8^+^CD161^int^ T_EM_ cells in PP-MS (**Supplementary Figure 4**), no changes in the frequency of CD8^+^CD161^high^ naïve T cells in MS (**Figure 2A**), but lower frequencies of CD8^+^CD161^high^ T_EM_ cells specifically in PP-MS patients and of CD8^+^CD161^high^ T_CM_ cells in PP-MS and SP-MS (**Figure 2A**). No significant differences in the frequencies of CD8^+^ CD161^high^ populations were detected in RR-MS and ALS (**Figure 2A**). The evaluation of all CD3^+^ CD161^high^ Vα7.2^+^ T cells highlighted a specific reduction of MAIT cells in PP-MS (**Supplementary Figures 5**, **6**). Further stratification of MAIT cells according to CD4 and CD8 markers did not evidence any unbalance in CD4 and/or CD8 expression under disease (**Supplementary Figures 5**, **7**). On the other hand, the analyses of the CD8^+^ T cells expressing high levels of CD161 and the invariant Vα7.2 TCR chain revealed no alterations in the frequency of MAIT cells, including their naïve, T_CM_ and T_EM_ fractions, in RR-MS and ALS (**Figure 2B**). A significant reduction of Vα7.2^+^CD161^high^ T_CM_ cells was detected in SP-MS samples, in line with the respective CD161^high^ population (**Figure 2B**). Naïve MAIT cells, scarcely present in the circulation of healthy subjects, were absent in PP-MS (**Figure 2B**). Most importantly, the abundance of total CD8^+^Vα7.2^+^CD161^high^ MAIT cells was significantly lower in PP-MS and this observation was paralleled by the drop in MAIT cells with a T_CM_ and T_EM_ phenotype (**Figure 2B**).

## Discussion

Here we show the selective reduction of CD8^+^ MAIT cells, characterized by high surface levels of CD161 and the expression of TCR invariant alpha chain Vα7.2 (14), in blood cells of patients affected by primary progressive MS and no other MS courses or another neurological disorder, such as ALS.

A previous paper from our group described transcriptional down-regulation of CD161 and loss of circulating CD8^+^CD161^high^ T cells in PP-MS but did not address disease specificity of the observation and neither clarified whether this reduction was due to the loss of MAIT cells in blood or rather to the downregulation of CD161, which may occur under chronic stimulation (18). Similarly, alterations in CD161^high^ T cell populations in MS described in other studies cannot undoubtedly refer to MAIT cells as lacking specific stainings (11, 19) or to progressive MS forms, as MS subjects with distinct disease courses were pooled in one cohort (19). In our study CD161 gene expression analysis was extended to RR-MS and SP-MS transcriptomics data, and MAIT cell frequency by flow cytometry was enriched by the detection of Vα7.2 on CD161^high^ CD8^+^ T cells in novel cohorts of untreated PP-MS subjects and MS patients with other disease courses thus allowing comparisons among distinct forms of MS.

Some reports suggest reduced circulating MAIT cell frequency in relapsing RR-MS (9, 20, 21) or describe controversial results for the remission phase of RR-MS using sample sizes similar to ours (20–22). In our study, RR-MS patients under remission did not display any alterations in expression levels of CD161 and other MAIT cell markers or in MAIT cell frequency in two distinct cohorts of diseased and control subjects. While MAIT cell reduction has been reported for pooled cohorts of PP-MS and SP-MS subjects (10), this observation is not confirmed by a study analyzing SP-MS subjects separately (22). In the present investigation lowering of circulating CD8^+^CD161^high^ T cells was specific to PP-MS and was mirrored in the respective CD45RO^+^CCR7^+^ central and CD45RO^+^CCR7^-^ effector memory fractions. The concomitant evaluation of Vα7.2 TCR invariant chain expression in CD8^+^CD161^high^ populations validated the hypothesis that the reduced frequency of CD8^+^CD161^high^ T cells was due to the drop of Vα7.2^+^ MAIT cells in PP-MS, while the minor shrinkage in central memory CD8^+^ MAIT cells in SP-MS was in line with the reduction of the respective CD161^high^ population but did not impact total CD8^+^ MAIT cells.

Recent literature for ALS reported about dysregulations in MAIT cell-related markers as identified by single cell RNA- and TCR-seq (12, 13). Notably, CD161 mRNA was upregulated in ALS (12, 13). Nevertheless, these transcriptomics data were relative to a few ALS patients and were not validated at protein and cellular level. In this regard, ALS blood was not characterized by any changes in the frequency of CD8^+^ MAIT cells but presented significant increases of CD4^+^CD161^+^ and CD8^+^CD161^int^ T cells, which may represent recently activated inflammatory T cells (23–26). Altogether, we provide novel relevant information about the definition of the immune set-up in ALS.

Concerning the possible role of MAIT cells in chronic inflammation, CD8^+^CD161^+^ T lymphocytes are reduced in blood during chronic infections or autoimmune diseases while enriched in infected or inflamed tissues (14, 27–29), suggesting their relocation under disease. The observations that MAIT cells may be higher in cerebrospinal fluid than peripheral blood in RR-MS during exacerbation (9) and that activated CD8^+^CD161^+^Vα7.2^+^ T cells are detected in active and chronic active lesions of secondary progressive MS cases (20) may indicate recruitment of circulating MAIT cells into the CNS. However, considering that active lesions are not peculiar for a single clinical disease course (30), the issue about MAIT cell entry into the CNS of patients with distinct disease courses should be comparatively investigated in PP-MS, SP-MS and RR-MS tissues.

Overall, our work shows a systematic analysis of circulating CD161^high^Vα7.2^+^CD8^+^ MAIT cells at distinct courses of MS and in ALS, and demonstrates that the drop of CD8^+^ MAIT cells, also in the respective T_EM_ and T_CM_ subpopulations, represents an immunological feature emerging in primary progressive disease. Considering that MAIT cell numbers in blood are highly variable under healthy conditions, whether the low frequency emerges under disease or is rather a precondition for primary progressive MS remains to be established. Finally, further investigations are needed for validation of this immunological parameter as clinical biomarker.

## Data availability statement

The datasets presented in this study can be found in online repositories. The names of the repository/repositories and accession number(s) can be found in the article/**Supplementary Material**.

## Ethics statement

The studies involving humans were approved by Ethics Committee of Ospedale San Raffaele. The studies were conducted in accordance with the local legislation and institutional requirements. The participants provided their written informed consent to participate in this study.

## Author contributions

DD: Writing – review & editing, Writing – original draft. CB: Writing – review & editing. RC: Writing – review & editing. FM: Writing – review & editing. AG: Writing – review & editing. FE: Writing – review & editing. NR: Writing – review & editing. AQ: Writing – review & editing. VM: Writing – review & editing. MF: Writing – review & editing. CF: Writing – review & editing, Writing – original draft.

## Supplementary material

Supplementary Figure 1 Demographics of subjects under analyses. Age distribution of female (F) and male (M) subjects in each group. The number of female and male healthy and diseased were reported over each distribution.

Supplementary Figure 2 Gating strategy for CD4^+^CD161^+^ T cells, CD8^+^CD161^int^ T cells, CD8^+^CD161^high^ T cells and CD8^+^ MAIT cells in a representative sample.

Supplementary Figure 3 Gene expression levels of MAIT cell-related markers in PBMC. Comparisons were performed for each disease group vs. the relative healthy control group. Group comparisons were evaluated by unpaired t-test with Welch’s correction in case of unequal variances or Mann-Whitney test in case of non-normal distribution. * *p*<0.05, ** *p*<0.01, *** *p*<0.001.

Supplementary Figure 4 Frequency of circulating CD4^+^ CD161^+^ **(A)** and CD8^+^ CD161^int^ **(B)** T cells in diseased and control groups. Group comparisons were evaluated by unpaired t-test with Welch’s correction in case of unequal variances or Mann-Whitney test in case of non-normal distribution. * *p*<0.05.

Supplementary Figure 5 Gating strategy for CD3^+^ CD161^high^ Vα7.2^+^ MAIT cells and relative CD4 and/or CD8 positive or double negative subsets in a representative sample.

Supplementary Figure 6 Frequency of CD161^high^ Vα7.2^+^ T cells among circulating CD3+ cells in diseased and control groups. Group comparisons were evaluated by unpaired t-test with Welch’s correction in case of unequal variances or Mann-Whitney test in case of non-normal distribution. * *p*<0.05.

Supplementary Figure 7 Frequency of CD4 and/or CD8 positive or double negative cells among CD3^+^ CD161^high^ Vα7.2^+^ T cells in diseased and control groups.
