# Supplementary material for: Circulating MAIT cells in multiple sclerosis and amyotrophic lateral sclerosis
Source: Front Immunol. 2024 Jul 23;15:1436717. doi: 10.3389/fimmu.2024.1436717 (PMC11300250; doi:10.3389/fimmu.2024.1436717)
Supplement: Supplementary Figure 1 — Demographics of subjects under analyses. Age distribution of female (F) and male (M) subjects in each group. The number of female and male healthy and diseased were reported over each distribution. [file DataSheet_1.pdf]

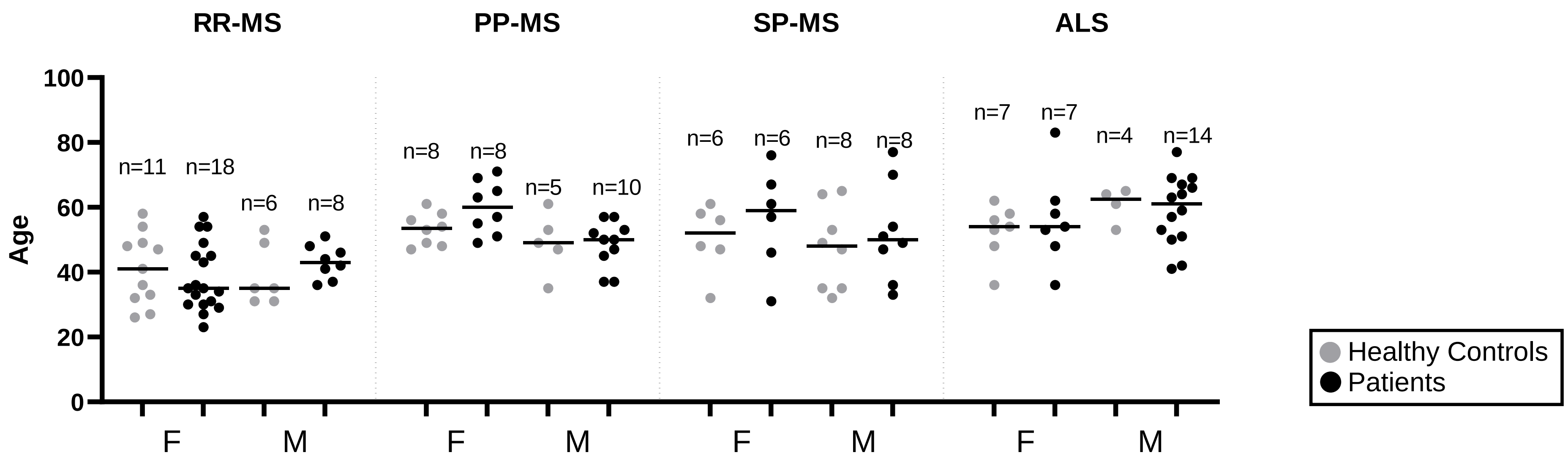

Supplementary Figure 1

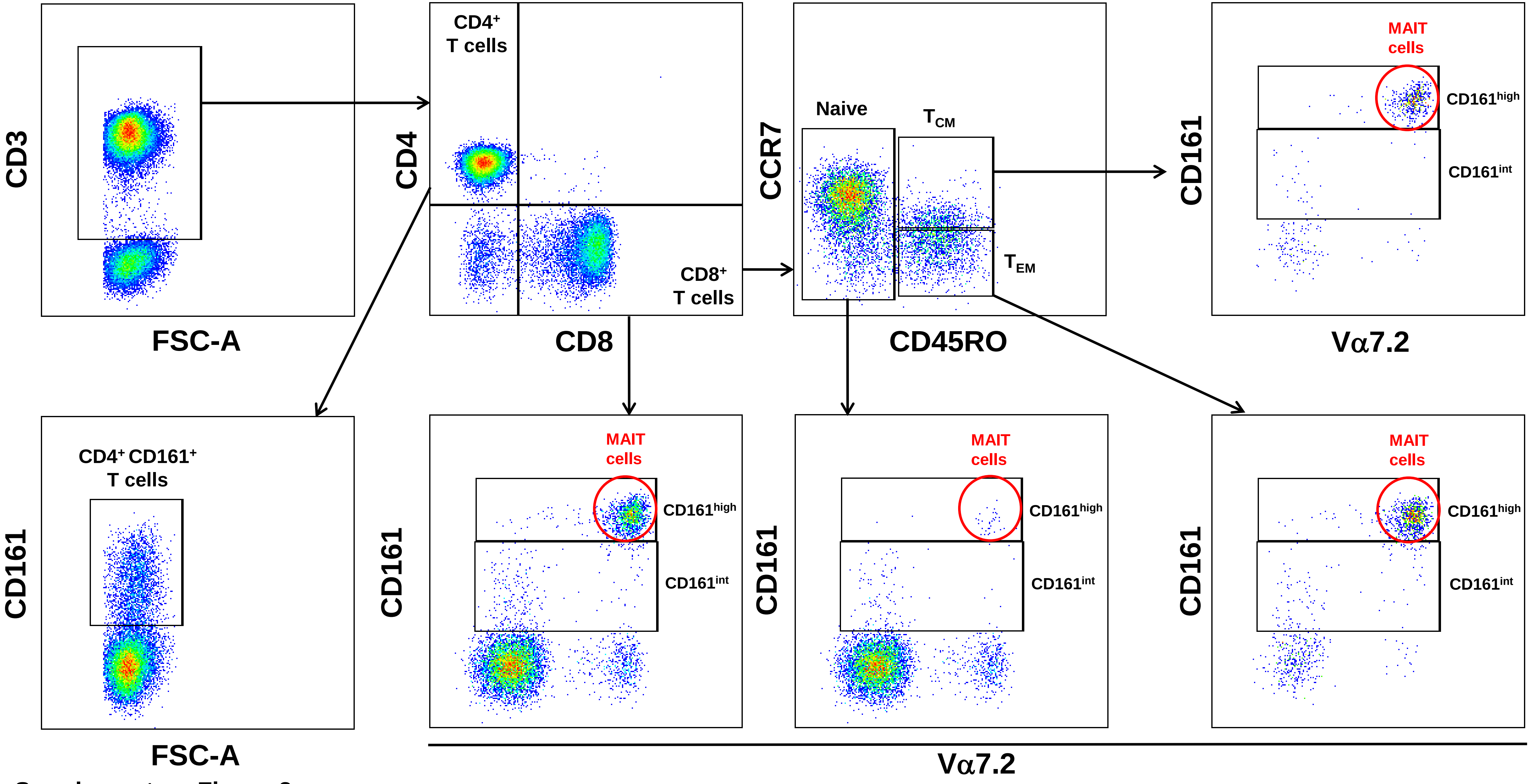

Supplementary Figure 2

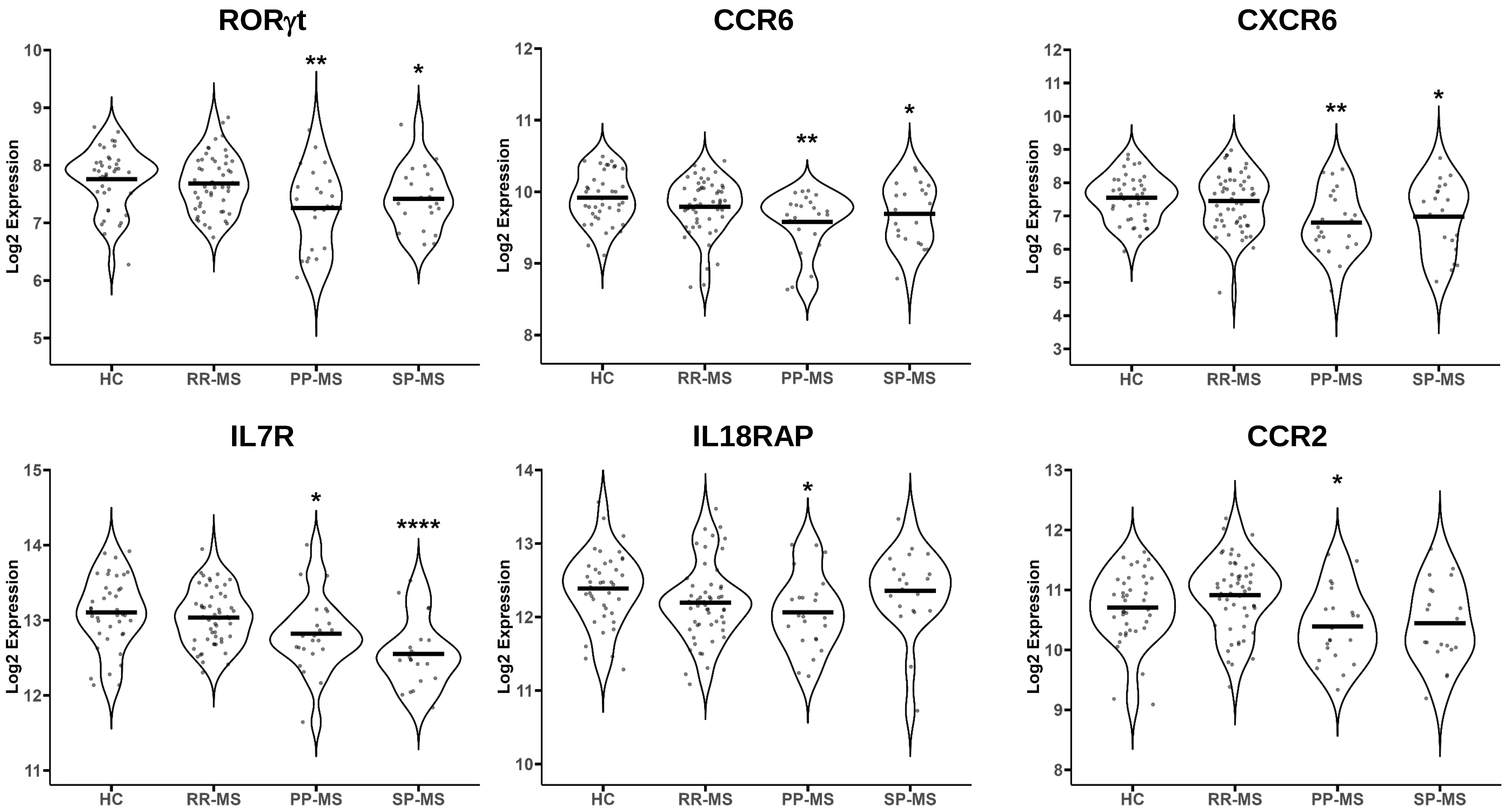

Supplementary Figure 3

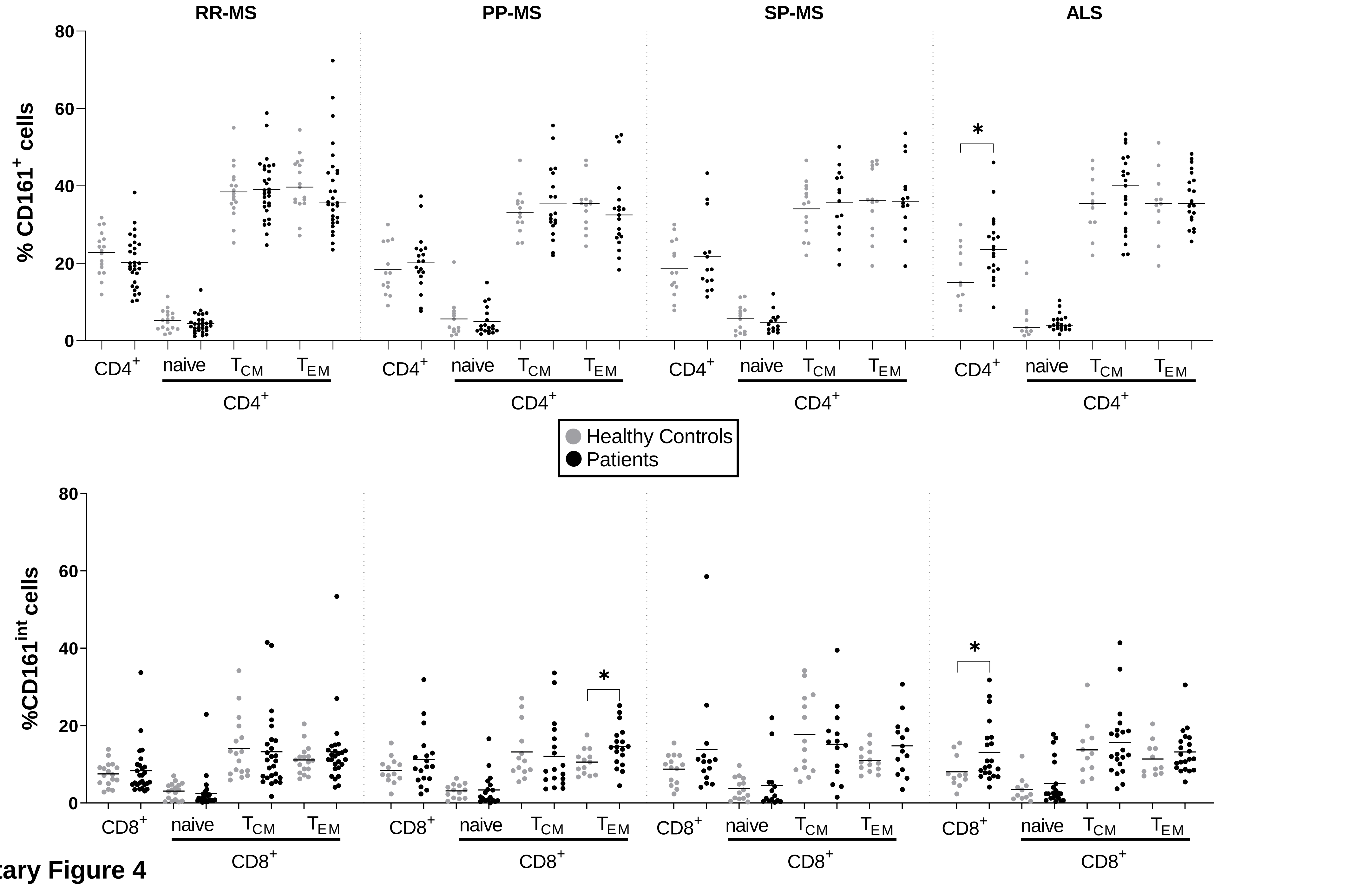

Supplementary Figure 4

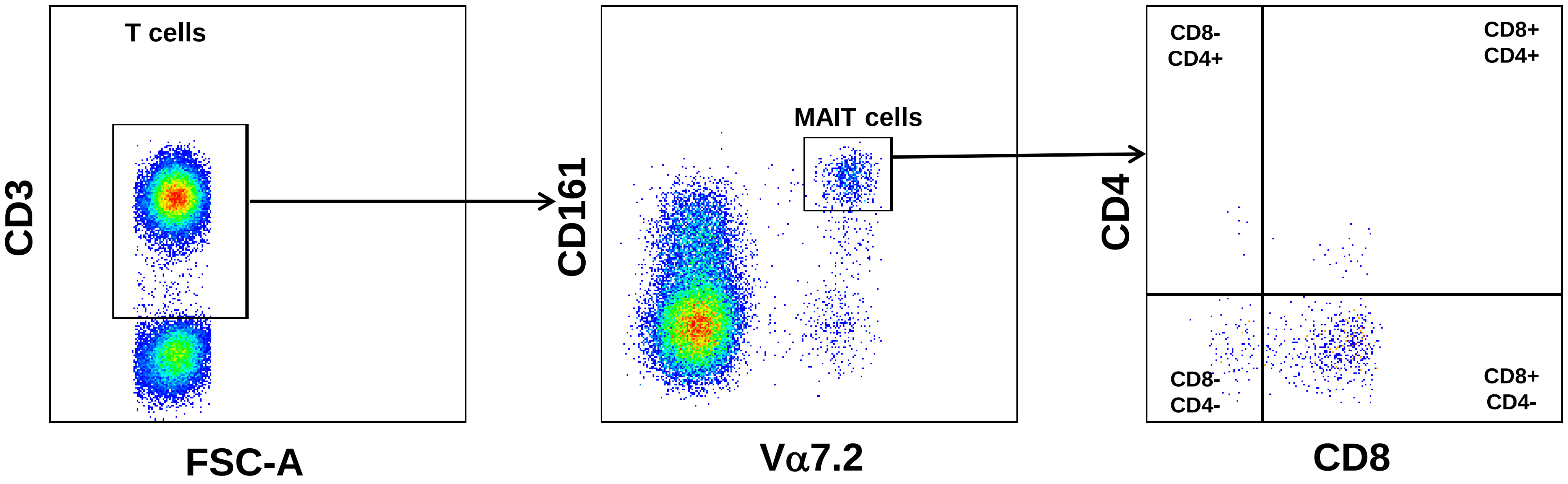

Supplementary Figure 5

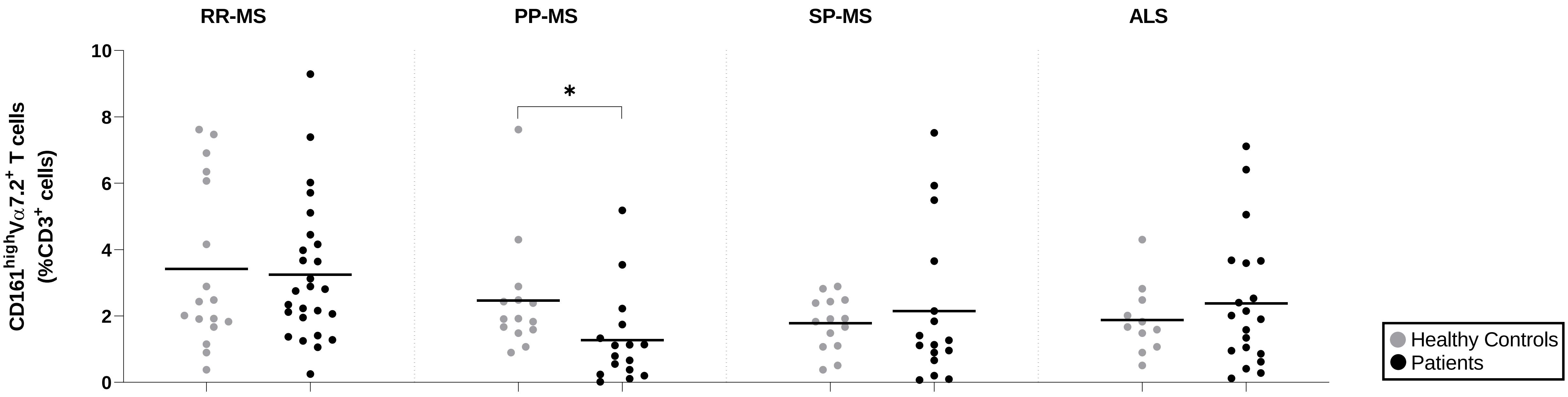

Supplementary Figure 6

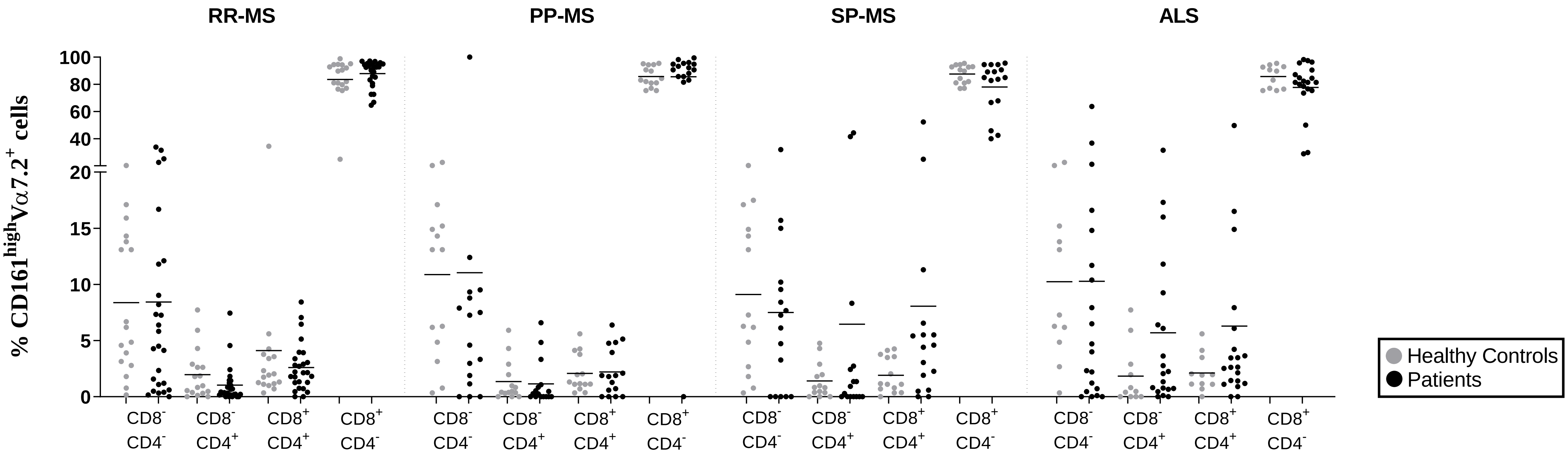

Supplementary Figure 7
